# Total Hip Arthroplasty for Avascular Necrosis in a Patient With Hemophilia B

**DOI:** 10.1016/j.artd.2024.101482

**Published:** 2024-10-12

**Authors:** Siddhartha Dandamudi, Joyee Tseng, John Ratz, Lisa Boggio, Brett R. Levine

**Affiliations:** a Rush University Medical Center, Chicago, IL, USA; b University of California Davis School of Medicine, Sacramento, CA, USA; c Loyola University, Chicago, IL, USA

**Keywords:** Hemophilia B, Hip arthroplasty, Case report, Bleeding management

## Abstract

Avascular necrosis (AVN) of the femoral head accounts for up to 10% of all total hip arthroplasties performed annually. Typically associated with intravascular coagulation, AVN is extremely rare in patients with bleeding disorders such as hemophilia B. In this report, we describe the therapeutic management of a 46-year-old male with hemophilia B, presenting with chronic left hip pain and AVN of the femoral head. He presented with progressive groin pain for 6 months and was unable to ambulate without the assistance of crutches. Radiographs showed evidence of degenerative joint disease secondary to AVN of the femoral head. After exhausting treatment options, the patient elected to proceed with an elective total hip arthroplasty. Intricate preoperative planning and hemophilia management were required by a multidisciplinary team to mitigate bleeding risks and promote success of the surgery. Postoperatively, the patient experienced a short-term rise in creatinine, but experienced no bleeding complications. The long-term follow-up revealed significant functional improvement without any complications of hemophilia B. There are no reports outlining AVN in hemophilia B (factor IX deficiency) or step-by-step treatment strategies for successful hip replacement in these patients.

## Introduction

Avascular necrosis (AVN) of the femoral head is characterized by a disruption of blood supply leading to collapse of the femoral head affecting 20,000 new Americans every year [1]. The disruption leading to AVN is typically associated with intravascular coagulation causing ischemia or traumatic injury to the femur [2,3]. A less common cause of AVN is extravascular compression due to bleeding [4]. Hemophilia B, affecting 1 in 30,000 births, is an X-linked recessive disorder categorized by a dysfunction or deficiency of factor IX resulting in clotting abnormalities. This consequently leads to excessive and repetitive bleeding episodes from synovial tissue/intra-articular structures, muscles, soft tissues, and mucosa [5,6]. Internal bleeding due to hemophilia B can lead to recurrent hemarthroses with subsequent hip joint destruction and, in rare instances, may cause AVN secondary to intraosseous hypertension within the femoral head [2,3,7]. It has been documented that hemophilia, while less common, may paradoxically cause AVN [7,8]. It is known that when the femoral head has collapsed due to AVN, total hip arthroplasty (THA) is the treatment of choice in hemophilia patients [[9], [10], [11]].

Hemophilia B patients with AVN are extremely rare; there is a dearth of literature on the management, operative treatment, and outcomes of THA in these patients. Most studies looking at THA in hemophilia patients do not have AVN as the indication for THA and focus on management and outcomes of hemophilia A patients with no commentary on patients with hemophilia B. While rates of total joint replacement have been shown to be the same in patients with hemophilia A and B, hemophilia B has a much lower incidence rate, and guidelines have established that factor IX has a longer half-life at 18-34 hours than factor VIII (8-12 hours), necessitating different management strategies perioperatively for these patients [7,12,13]. Reported outcomes for the small number of hemophilia patients undergoing THA have overall been positive, but no descriptions of the diagnostic and therapeutic journey of hemophilia B patients with AVN have been written. This case effectively outlines how to perform THA in such patients successfully without bleeding complications through effort from a multidisciplinary team of a hematologist and orthopaedic surgeon.

## Case history

A 46-year-old Hispanic male with a history of moderate hemophilia B diagnosed at 8 years old presented to our institution’s emergency department in November 2022 with complaints of left hip pain. The patient was seen by an outside orthopaedic surgeon who diagnosed him with AVN of the femoral head. The outside surgeon was not comfortable operating on him and referred him to our institution for a second opinion.

Historically, the patient was diagnosed with hemophilia B following multiple episodes of epistaxis as a child. He started frozen plasma infusions that continued for 23 years. He experienced 2 to 3 major joint bleeds a year during this time. He was then switched to recombinant extended half-life factor IX 500 units weekly about 4 years ago. His last joint bleed was in his right knee in June 2022, requiring several factor infusions and hospitalization for 1 week.

In February 2023, the patient established care with our institution’s hematology department. The hematologist determined that his recombinant factor IX dosage was too low as his factor IX level was 3% (50%-100% normal range). His medication was adjusted to 3500 units (50 IU/kg per day) of extended half-life recombinant factor IX weekly to keep his trough level of factor IX activity above 1%-5%.

In March 2023, the patient presented to our institution’s orthopaedic clinic for progressive pain in his left groin and hip that had progressively worsened over 6 months. The pain was described as constant, tight, and worse with movement. Symptoms also included severe weakness and the inability to ambulate without crutches. On physical examination, he had a severe limp on the affected leg, a positive Trendelenburg sign, reduced abductor strength, a shortened left leg, and maximum flexion of 90 degrees of the left hip. At that time, the patient had a body mass index of 24.2 kg/m^2^.

**Figure 1:**
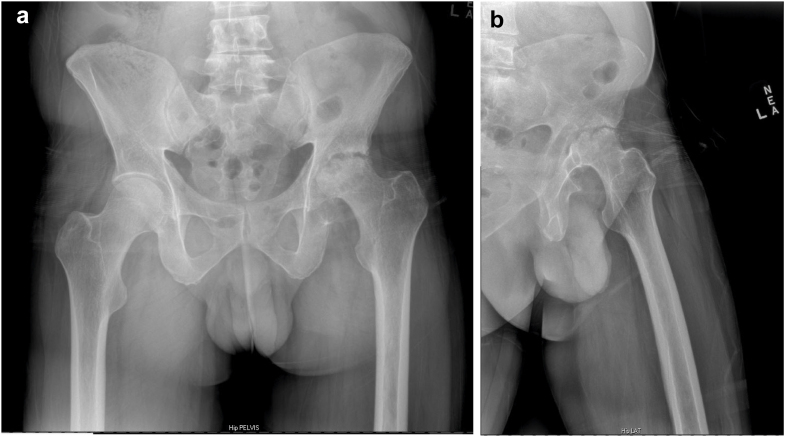
(a) Anteroposterior pelvis X-ray of the left hip showing avascular necrosis of the femoral head. (b) Lateral X-ray of the left hip showing avascular necrosis of the femoral head.

Our independent evaluation of the radiographs showed severe AVN of the femoral head and secondary bone-on-bone arthritis. The femoral head had migrated superiorly and eroded into the acetabulum with a loss of the superior aspect of the femoral head (Fig. 1a and b). With radiographic evidence of degenerative joint disease and clinical evidence of his inability to ambulate or perform his activities of daily living, he was indicated for a left THA. Due to his higher risk of bleeding from the hemophilia B, careful planning with his hematologist for preoperative and postoperative management of factor replacement was necessary. Prior to the surgery, the patient’s hematologist created a plan for his recombinant factor IX dosing as well as guidelines for pain management perioperatively:

1. 100 IU/kg (6500 units) ± 10% preoperatively 30-60 minutes to get to 100% factor IX level
2. Postoperative 50 IU/kg (3250 units) ± 10% every 24 hours until discharge to keep trough of factor IX activity above 50%
3. 50 IU/kg (3000 units) every other day for 3 doses after discharge to keep trough of factor IX activity above 30%
4. Back to weekly dosing of 50 IU/kg (3500 units) after 1 week
5. Avoid NSAIDs for postoperative pain or fever due to increased bleeding risk
6. No anticoauglation postoperatively due to low factor IX levels.

## Surgical procedure

The patient was cleared medically for surgery. His preoperative hemoglobin was 13.2 g/dL. The patient was given 1 g of tranexamic acid (TXA) intravenously along with the 6500 units of recombinant factor IX prior to surgery. He received a general anesthesia for the surgery.

**Figure 2:**
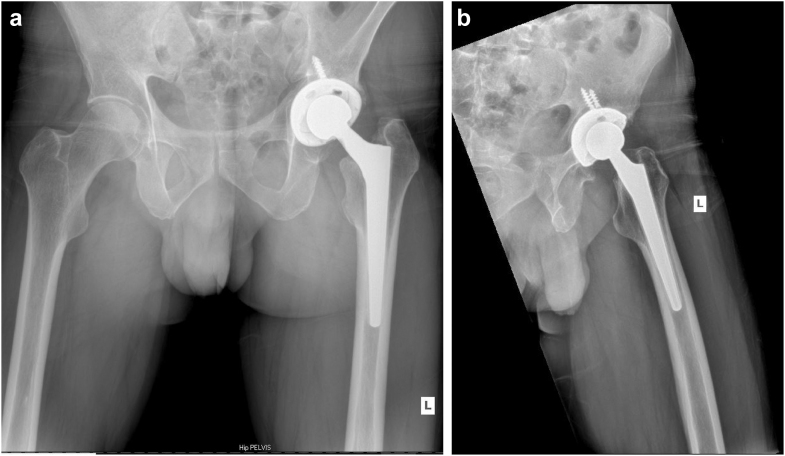
(a) AP pelvis X-ray of the left hip showing good alignment of the implants. (b) Lateral X-ray of the left hip showing good alignment of the implants.

The THA was performed via a standard posterior approach without any complications and an estimated intraoperative blood loss of 300 mL. Based on a preoperative template the patient received a Wagner Cone femoral component and a G7 acetabular implant (Zimmer-Biomet, Warsaw, IN). A ceramic-on-polyethylene articulation was used with a 32-mm biolox delta femoral head (CeramTec, Plochingen, Germany). A standard wound closure was performed with topical skin adhesive and sterile dressing being placed. The pathology report did not describe any AVN of the bone but did describe severe degenerative joint disease. The lack of AVN on pathology may have occurred as a large piece of the femoral head was completely missing at the time of surgery. Postoperative radiographs showed good alignment of the implants (Fig. 2a and b).

## Postoperative outcome

The patient spent 5 days in the hospital postoperatively and did not require a blood transfusion. He did have a minor bump in creatinine from his baseline of 1.47 mg/dL to 1.88 mg/dL that resolved with fluids. His postoperative hemoglobin was 9.8 but stabilized to 8.6 g/dL by discharge. The patient’s factor IX activity was 38.8% on postoperative day 2 and 39.4% on discharge. He ultimately passed his physical and occupational therapy goals, was cleared medically, and was discharged home with no acute complications. His early postoperative course was uncomplicated, and he completed a short course of home and outpatient physical therapy without difficulty.

At his 6-month follow-up visit, he was very satisfied with no reported complications or hospitalizations. He had marked improvement in his symptoms and full return to his activities of daily living. Preoperatively, he rated his pain a 6/10, and postoperatively, he reported being pain-free. His Harris hip score (HHS) improved from 29 preoperatively to 89 at this visit. He reported he did not experience any bleeding episodes, changes in his hemophilia B management, or hospitalizations. On physical examination, the patient had a well-healed, dry incision with no erythema or drainage and was able to ambulate without any assistive devices. He had improved abductor strength, a negative Trendelenburg sign, and a maximum flexion of 100 degrees in his left hip. Radiographic evaluation showed well-placed components with no evidence of subsidence, migration, wear, radiolucent lines, or loosening (Fig. 2).

## Discussion

Up to 10% of all THAs performed are due to AVN, yet the etiology is rarely attributed to bleeding disorders in a patient. Only one report from Paton et al. speaks about 3 cases of AVN of the femoral head in hemophilia patients from one center [14]. No other reports exist on AVN of the femoral head in hemophilia patients, but the association as a potential etiology has been mentioned in articles discussing AVN [7,8,15]. Hemophilia patients have a higher incidence of knee, elbow, and ankle arthropathy compared to the hip. The sequela of hip arthropathy-leading and secondary AVN is very rare in hemophilia B patients [16].

Indications for THA due to AVN in hemophilic patients are similar to the rest of the population, which includes limited joint function following conservative therapies along with severe, debilitating pain during movement [10,17]. For this patient, proper management of bleeding risks preoperatively and a plan with prophylaxis of factor IX intraoperatively allowed for a successful THA treatment option for this patient [12,18].

Hemophilia poses difficulties in the perioperative period for the surgeon due to the immense risk of bleeding. Guidelines have been established on perioperative factor levels to minimize bleeding risk for patients undergoing THA, but no literature has outlined a case of a hemophilia B patient in practice [6,12,18,19]. Prior to surgery, Wiedel et al. state that ideally factor IX should be corrected to 120%, but “the range of half-life of the purified and recombinant factor IX products is extremely variable from patient to patient” [12,17]. A case series done in Ireland of 11 patients (9 hemophilia A and 2 hemophilia B) showed a mean blood loss of 502 mL compared to the established normal of 400 mL [20]. A separate study of 21 hemophilia patients (1 hemophilia B) showed an average blood loss of 721 mL intraoperatively [16].

In this case, the patient lost significantly less blood than reported in prior studies at 300 mL [16,20]. The combination of a preoperative plan to administer recombinant factor IX along with intraoperative use of TXA most likely contributed to this low intraoperative blood loss. Intraoperatively, Huang et al. showed that in 34 patients with hemophilia A who underwent 24 total knee arthroplasty and 18 THA, the use of tranexamic acid decreased perioperative blood loss, transfusion rate, and supplemental amount of factor needed [21]. Wu et al. reported in their case series of 21 hemophilia patients that an average of 14,031 units (range 8000-25,200) of factor was used in the hemophilia A patients, and 17,600 units of factor were used in the hemophilia B patient intraoperatively [16]. Our patient used 6500 units (100 IU/kg) of recombinant factor IX preoperatively, which is lower than any of the patients in the case series. TXA proved valuable in controlling intraoperative bleeding for the patient [21,22].

Postoperatively, the hemoglobin did drop by 4.6 g/dL from his preoperative start over his 5-day stay in the hospital, which is slightly more than average but reasonable [23]. Published literature shows an expected drop of an average of 3.25 g/dL in 48 hours for patients undergoing THA with hemophilia [20]. Part of his drop in hemoglobin may be explained by the continuous fluids he received inpatiently for his minor bump in creatinine. Evidence shows us that administration of 500 mL of fluids may decrease hemoglobin 1 g/dL [24]. The patient in this case was deemed stable clinically and did not require blood transfusions.

The patient’s postoperative course was promising. His improvement in range of motion and HHS is consistent with reports of patients with hemophilia in the literature. Wu et al. commented on the outcomes of 24 THA in 20 hemophilia A and 1 hemophilia B patients over an average of 113 months of follow-up, stating 100% survivorship, no complications, and improvement of HHS from 37 preoperatively to 90 at last follow-up [16]. Lee et al. described a similar increase in 27 hips of HHS from 57 preoperatively to 94 postoperatively [25]. Proper management of bleeding risk and continuity of care postoperatively ensure improvement both short and long term.

It has been described that prophylaxis in hemophilia patients via factor therapy is the most effective way to avoid joint destruction in patients [26]. However, even with therapy, the 12-month prevalence of hemarthrosis episodes in adult hemophilia B patients is 42% [27]. Clinical follow-up can lengthen the time before surgery, but THA will continue to be a mainstay of treatment for those patients with joint destruction due to hemarthrosis leading to AVN.

## Summary

This case report outlines a rare case of AVN in a hemophilia B patient and outlines an effective surgical plan to ensure a successful THA. This is also the first report following a patient that is using extended half-life factor IX to control bleeding episodes perioperatively. The collaboration among interdisciplinary steams to minimize the bleeding risk of the patient perioperatively is described, and outcomes have been encouraging.

## Conflicts of interest

L. Boggio is a paid employee for Rush University Medical Center; is a paid consultant for Novo Nordisk, OctaPharma, Sanofi, Genentech, Bayer, and Pfizer; receives research support from Sanofi, Octapharma, Bayer, Sanofi, and Genentech; is an exam approval committee member of the American Board of Internal Medicine; is an education committee member of the International Society of Thrombosis and Haemostasis; and is a member of the Hemostasis and Thrombosis Research Society, Grant review committee, Women with thrombosis and thrombophilia committee, and Device related thrombus committee. B. R. Levine receives IP royalties from Link Orthopedics; is a paid consultant for Enovis, Link Orthopedics, Merete, and Zimmer Biomet; receives research support from Zimmer Biomet; is an editorial board member of Arthroplasty Today, Elsevier, Human Kinetics, SLACK Incorporated, and Wolters Kluwer Health-Lippincott Williams & Wilkins; and is a board/committee member of AAOS, American Association of Hip and Knee Surgeons, Knee Society, and MAOA. All other authors declare no potential conflicts of interest.

For full disclosure statements refer to https://doi.org/10.1016/j.artd.2024.101482.

## Informed patient consent

The author(s) confirm that written informed consent has been obtained from the involved patient(s) or if appropriate from the parent, guardian, power of attorney of the involved patient(s); and, they have given approval for this information to be published in this case report (series).

## CRediT authorship contribution statement

**Siddhartha Dandamudi:** Conceptualization, Data curation, Investigation, Project administration, Writing – original draft, Writing – review & editing. **Joyee Tseng:** Conceptualization, Methodology, Writing – review & editing. **John Ratz:** Conceptualization, Writing – original draft, Writing – review & editing. **Lisa Boggio:** Conceptualization, Investigation, Supervision, Validation, Writing – review & editing. **Brett R. Levine:** Conceptualization, Investigation, Methodology, Validation, Visualization, Writing – original draft, Writing – review & editing.

## Appendix A Supplementary Data

Conflict of Interest Statement for Boggio

Conflict of Interest Statement for Levine

Conflict of Interest Statement for Dandamudi

Conflict of Interest Statement for Ratz

Conflict of Interest Statement for Tseng
