# Supplementary material for: Total Hip Arthroplasty for Avascular Necrosis in a Patient With Hemophilia B
Source: Arthroplast Today. 2024 Oct 12;30:101482. doi: 10.1016/j.artd.2024.101482 (PMC11736053; doi:10.1016/j.artd.2024.101482)
Supplement: Conflict of Interest Statement for Boggio [file mmc1.pdf]

CONFLICT OF INTEREST STATEMENT

American Association of Hip and Knee Surgeons

(Adopted from the American Academy of Orthopaedic Surgeons disclosure statement)

The following form **must be filled out completely and submitted by each author (example, 6 authors, 6 forms). All items require a response. If there is no relevant disclosure for a given item, enter "*None.*"**

Case Report: Total Hip Arthroplasty for Osteonecrosis in a Patient with Hemophilia B

Manuscript Title

1. Royalties from a company or supplier (The following conflicts were disclosed)none

2. Speakers bureau/paid presentations for a company or supplier (The following conflicts were disclosed)None

3A. Paid employee for a company or supplier (The following conflicts were disclosed)
Rush University Medical Center

3B. Paid consultant for a company or supplier (The following conflicts were disclosed)
Novo Nordisk, OctaPharma, Sanofi, Genentech, Bayer, Pfizer

3C. Unpaid consultants for a company or supplier (The following conflicts were disclosed)
none

4. Stock or stock options in a company or supplier (The following conflicts were disclosed)
none

5. Research support from a company or supplier as a Principal Investigator (The following conflicts were disclosed)
Sanofi, Octapharma, Bayer, Sanofi, Genentech

6. Other financial or material support from a company or supplier (The following conflicts were disclosed)
none

7. Royalties, financial or material support from publishers (The following conflicts were disclosed)
none

8. Medical/Orthopaedic publications editorial/governing board (The following conflicts were disclosed)
none

9. Board member/committee appointments for a society (The following conflicts were disclosed)
American Board of Internal Medicine, Exam approval committee
International Society of Thrombosis and Haemostasis, Education committee
Hemostasis and Thrombosis Research Society
- Grant review committee
- Women with thrombosis and thrombophilia committee
- Device related thrombus committee

Each author must sign AND print or type his/her name, date and submit a separate form

In addition, one BLINDED Conflict of Interest form (no author names used) should be submitted per manuscript with all author disclosures.

Lisa N Boggio	*Lisa N Boggio*	4/15/2024
Author Name (Print or Type)	Author Signature	Date
