# Supplementary material for: Total Hip Arthroplasty for Avascular Necrosis in a Patient With Hemophilia B
Source: Arthroplast Today. 2024 Oct 12;30:101482. doi: 10.1016/j.artd.2024.101482 (PMC11736053; doi:10.1016/j.artd.2024.101482)
Supplement: Conflict of Interest Statement for Levine [file mmc2.pdf]

CONFLICT OF INTEREST STATEMENT

American Association of Hip and Knee Surgeons

(Adopted from the American Academy of Orthopaedic Surgeons disclosure statement)

Case Report: Total Hip Arthroplasty for Osteonecrosis in a Patient with Hemophilia B

Manuscript Title

1. Royalties from a company or supplier (The following conflicts were disclosed)

   Link Orthopedics: IP royalties

2. Speakers bureau/paid presentations for a company or supplier (The following conflicts were disclosed)

   None

3A. Paid employee for a company or supplier (The following conflicts were disclosed)

   None

3B. Paid consultant for a company or supplier (The following conflicts were disclosed)

   Enovis: Paid Consultant
   Link Orthopedics: Paid Consultant
   Merete: Paid Consultant
   Zimmer Biomet: Paid Consultant

3C. Unpaid consultants for a company or supplier (The following conflicts were disclosed)

   None

4. Stock or stock options in a company or supplier (The following conflicts were disclosed)

   None

5. Research support from a company or supplier as a Principal Investigator (The following conflicts were disclosed)

   Zimmer Biomet: Research Support

6. Other financial or material support from a company or supplier (The following conflicts were disclosed)

   None

7. Royalties, financial or material support from publishers (The following conflicts were disclosed)
   None

8. Medical/Orthopaedic publications editorial/governing board (The following conflicts were disclosed)

   Arthroplasty Today: Editorial or Governing Board
   Elsevier: Editorial or governing board
   Human kinetics: Editorial or governing board
   SLACK Incorporated: Editorial or governing board
   Wolters Kluwer Health - Lippincott Williams & Wilkins: Editorial or governing board

9. Board member/committee appointments for a society (The following conflicts were disclosed)

   AAOS: Board or Committee Member
   American Association of Hip and Knee Surgeons: Board or Committee Member
   Knee Society: Board or Committee Member
   MAOA: Board or Committee Member

Each author must sign AND print or type his/her name, date and submit a separate form

In addition, one BLINDED Conflict of Interest form (no author names used) should be submitted per manuscript with all author disclosures.

Brett Levine		04/01/2024
Author Name (Print or Type)	Author Signature	Date
